# Emergence of Plasmid-Mediated Resistance Genes *tet*(X) and *mcr-1* in Escherichia coli Clinical Isolates from Pakistan

**DOI:** 10.1128/mSphere.00695-21

**Published:** 2021-08-25

**Authors:** Ruichao Li, Mashkoor Mohsin, Xiaoyu Lu, Sabahat Abdullah, Asim Munir, Zhiqiang Wang

**Affiliations:** a Jiangsu Co-Innovation Center for Prevention and Control of Important Animal Infectious Diseases and Zoonoses, College of Veterinary Medicine, Yangzhou University, Yangzhou, Jiangsu, People’s Republic of China; b Institute of Comparative Medicine, Yangzhou University, Yangzhou, Jiangsu, People’s Republic of China; c Institute of Microbiology, University of Agriculture, Faisalabad, Pakistan; Antimicrobial Development Specialists, LLC

**Keywords:** *Escherichia coli*, *tet*(X), *mcr-1*, plasmids, clinical settings

## Abstract

The emergence of *tet*(X) represents a significant threat to human health. In this study, we aimed to investigate the genomic characterizations of *tet*(X)-positive clinical Escherichia coli isolates and provide genomic insight into the dissemination of antibiotic-resistant bacteria in clinical settings. Four *tet*(X)-positive E. coli isolates, PK5074, PK5086, PK5095, and PK5097, from 100 human clinical isolates were identified by PCR and were resistant to tigecycline. *tet*(X) genes were in IncFII plasmids in 4 E. coli isolates. Worryingly, PK5074 also carried an *mcr-1*-bearing IncHI2 plasmid. Notably, a relatively high cotransfer frequency of *tet*(X) and *mcr-1* in PK5074 was found. PK5086, PK5095, and PK5097 were categorized into sequence type 410 (ST410) and indicated clonal dissemination of *tet*(X)-positive strains in hospitals, but *tet*(X)-bearing plasmids in PK5086, PK5095, and PK5097 were nontransferable. We present the first report of clinical E. coli isolates harboring *tet*(X) in South Asia. Our results support the implication of humans as a potential reservoir for *tet*(X)-harboring E. coli. We provide insight into the dissemination of *tet*(X) and *mcr-1* in a clinical setting and highlight the current transmission of both critical resistance genes globally.

**IMPORTANCE** Global transmission of plasmid-mediated tigecycline resistance gene *tet*(X)-bearing Escherichia coli strains incurs a public health concern. However, the research focusing on the prevalence of *tet*(X)-positive isolates in clinical specimens is still rare, and to our knowledge, there is no such report from South Asia. Here, we characterized four E. coli clinical isolates harboring *tet*(X) of human origin in Pakistan and demonstrated clonal dissemination of *tet*(X)-positive isolates in hospitals. We report the emergence of an *mcr-1*-bearing IncHI2 plasmid together with a *tet*(X)-positive IncFII plasmid in one clinical isolate. Cotransfer of *tet*(X)- and *mcr-1-*carrying plasmids is worrying and warrants further investigations.

## OBSERVATION

Tigecycline is used as the last-resort antibiotic to treat infections caused by extensively drug-resistant (XDR) bacteria, particularly carbapenem-resistant *Enterobacteriaceae* (1, 2). However, the emergence of the plasmid-mediated tigecycline resistance gene *tet*(X4), the most prevalent *tet*(X) variant that confers high-level tigecycline resistance in Escherichia coli, represents significant threats to human health (3, 4). Recently, reports on *tet*(X) in *Enterobacteriaceae* isolates from humans have increased significantly, mainly from China and Singapore (4, 5). Here, we report the first identification of E. coli clinical isolates harboring *tet*(X) of human origin in Pakistan and characterize the genetic environment of *tet*(X). We also describe a relatively high cotransfer frequency of *tet*(X) and *mcr-1* in clinical isolate PK5074, which highlights the current worldwide transmission of both critical resistance genes.

Among 100 human clinical isolates, four *tet*(X)-positive isolates (including PK5074, PK5086, and PK5097 from hospital A and PK5095 from hospital B) were acquired, and they were identified as E. coli (Table 1). Antimicrobial susceptibility testing revealed that all 4 E. coli isolates conferred high-level resistance to tigecycline with the MICs ranging from 32 to 64 mg/liter. Worryingly, mobile colistin resistance gene *mcr-1* was also detected in strain PK5074 (Table 2). MICs for PK5074 revealed that E. coli PK5074 exhibited resistance to tigecycline, colistin, kanamycin, doxycycline, ampicillin, enrofloxacin, streptomycin, amoxicillin, florfenicol, terramycin, and tetracycline (Table 1). PK5086, PK5095, and PK5097 exhibited the same resistance spectrum and were resistant to tigecycline, gentamicin, kanamycin, doxycycline, ampicillin, enrofloxacin, ceftiofur, streptomycin, amoxicillin, ceftriaxone, florfenicol, terramycin, and tetracycline (Table 1). Four *tet*(X)-positive isolates were multidrug-resistant (MDR) bacteria. PCR and Sanger sequencing confirmed the *tet*(X) present in four strains was *tet*(X4), which is annotated as *tet*(X) in the following context.

**TABLE 1:** MICs of four *tet*(X)-carrying clinical E. coli isolates investigated in this study

Strain ID	Date of sampling (yr-mo-day)	MICs (mg/liter)^*a*^															
		GEN	KAN	DOX	AMP	ENR	CFF	STR	AMX	RIF	CEF	FFC	MEM	CST	TER	TET	TIG
PK5074	2019-12-17	1	>128	64	>128	64	1	128	>128	>512	≤0.125	>128	≤0.125	4	>128	128	64
PK5086	2019-12-23	>128	64	64	>128	>128	>128	>128	>128	8	>128	>128	≤0.125	≤0.125	>128	>128	32
PK5095	2020-03-01	128	128	64	>128	128	>128	>128	>128	8	>128	>128	≤0.125	≤0.125	>128	>128	32
PK5097	2020-07-01	128	64	64	>128	>128	>128	>128	>128	8	>128	>128	≤0.125	0.25	>128	>128	32
ATCC 25922		0.25	2	0.5	4	≤0.125	≤0.125	4	4	4	≤0.125	4	≤0.125	0.25	4	0.5	≤0.125

a GEN, gentamicin; KAN, kanamycin; DOX, doxycycline; AMP, ampicillin; ENR, enrofloxacin; CFF, ceftiofur; STR, streptomycin; AMC, amoxicillin; RIF, rifampicin; CEF, ceftriaxone; FFC, florfenicol; MEM, meropenem; CST, colistin; TER, terramycin; TET, tetracycline; TIG, tigecycline.

**TABLE 2:** Genomic information of the chromosomes and plasmids of E. coli PK5074 and PK5086 resolved by hybrid assembly strategy

Strain	MLST	Components	Size (bp)	Accession no.	Replicon type(s)	Resistance genes	Virulence-associated gene(s)
PK5074	ST48	PK5074-chromosome	4,746,945	CP072802		*mdf*(A), *dfrA1*	*gad*, *ompT*, *terC*
		pPK5074-MCR1	267,744	CP072803	IncHI2, IncHI2A	*mcr-1*, *aph(3′)-Ia*, *aadA8*, *lnu*(F), *sul3*, *tet*(A), *aph(6)-Id*, *floR*, *ARR-2*, *dfrA14*	*terC*
		pPK5074-tetX	110,313	CP072807	IncFII	*tet*(X), *aph(3′)-Ia*, *aph(6)-Id*, *aadA22*, *aph(3′')-Ib*, *sul2*, *floR*, *bla*_TEM-215_	*traT*
		pPK5074-91kb	91,224	CP072806	IncFIB(K)	*qnrS2*, *tet*(A), *floR*, *sul2*, *aph(6)-Id*, *aph(3′')-Ib*	ND^*a*^
		pPK5074-69kb	69,302	CP072804	IncY	*qnrS1*, *aph(6)-Id*, *aph(3′')-Ib*, *aadA2*, *aph(3′)-Ia*, *bla*_TEM-1B_, *lnu*(F)	ND
		pPK5074-2kb	1,943	CP072805	ColRNA1	None	ND
PK5086	ST410	PK5086-chromosome	4,781,220	CP080370		*mdf*(A), *bla*_CMY-2_	*gad*, *lpfA*, *terC*
		pPK5086-tetX	100,261	CP080371	IncFII	*tet*(X), *fosA4*, *mph*(A), *dfrA12*, *floR*, *bla*_TEM-215_	*traT*
		pPK5086-97kb	97,614	CP080372	IncFIB (AP001918), IncFIA, IncQ1, IncFII (pRSB107)	*aac(6′)-Ib-cr*, *aph(3′')-Ib*, *aph(6)-Id*, *aadA5*, *aac(3)-IId*, *mph*(A), *dfrA17*, *sul1*, *sul2*, *tet*(B), *bla*_TEM-1B_, *bla*_OXA-1_, *bla*_CTX-M-15_	ND
		pPK5086-95kb	95,348	CP080373	IncY	None	ND
		pPK5086-2kb	2,088	CP080374	Col (BS512)	None	ND

a ND, not detected.

To investigate the transferability of *tet*(X) or *mcr-1*, conjugation assays were performed. Resistance genes *tet*(X) and *mcr-1* in strain PK5074, with corresponding resistance phenotypes for tigecycline and colistin, were able to successfully transfer from E. coli PK5074 into the recipient E. coli J53, suggesting that the *tet*(X) and *mcr-1* genes were located in conjugative plasmids or other mobilizable genetic elements in PK5074. The *tet*(X)-positive genetic structure exhibited good transferability into E. coli J53 at a frequency of (4.34 ± 0.07) × 10^−1^ cells per recipient. Comparatively, the *mcr-1*-bearing genetic structure transferred with a frequency of (6.46 ± 0.82) × 10^−6^ cells per recipient. Cotransfer of *tet*(X) and *mcr-1* was at a frequency of (6.18 ± 0.99) × 10^−6^ cells per recipient. However, *tet*(X) in strains PK5086, PK5095, and PK5097 was nontransferable.

**FIG 1:**
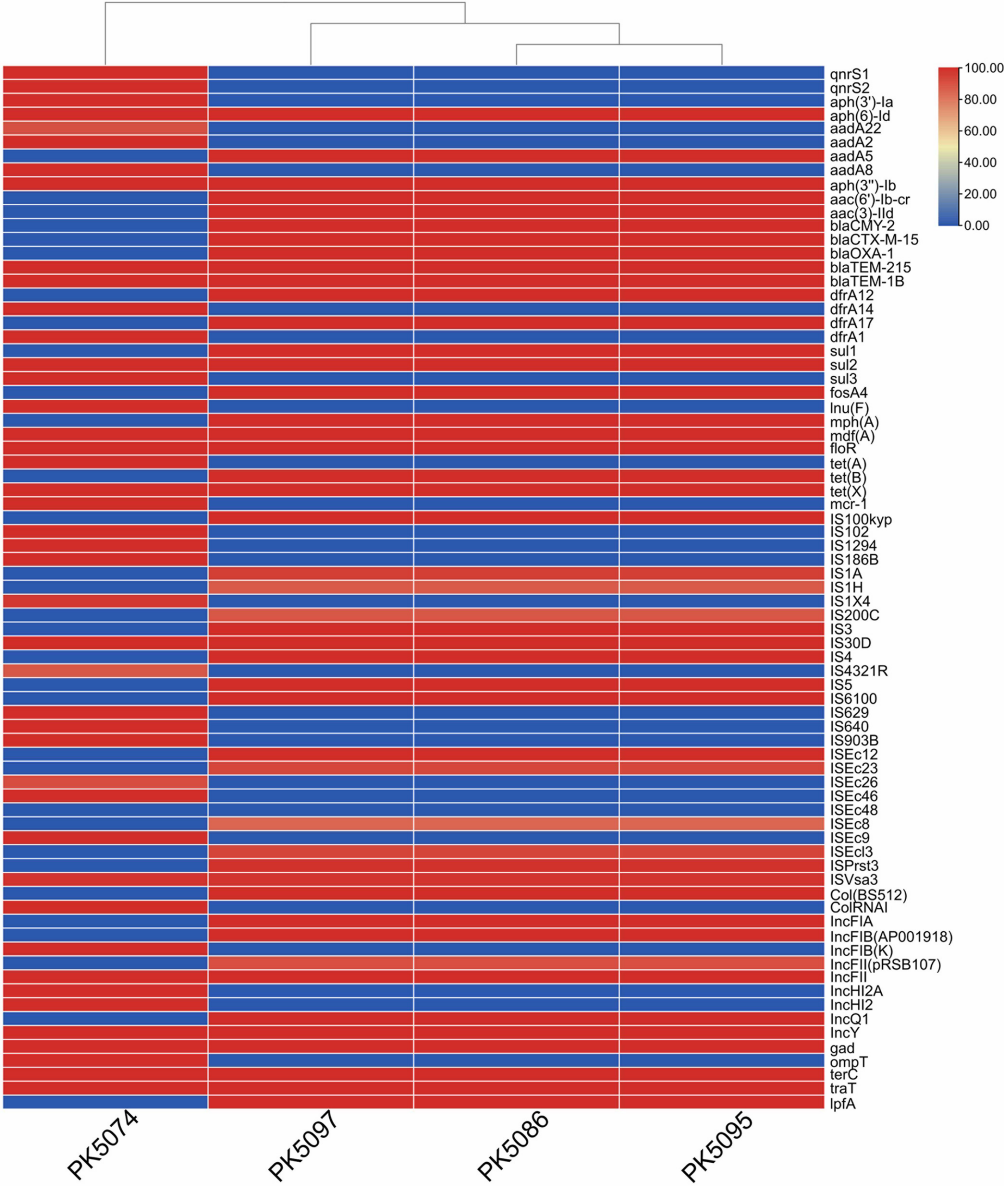
Distributions of antimicrobial resistance genes, insertion sequences, virulence-associated genes, and plasmid replicons in four *tet*(X)-bearing isolates. The color in figure legend indicates the percentage of sequence homology.

All the 4 *tet*(X)-carrying isolates were sequenced using the Illumina HiSeq 2500 platform generating 2 × 150-bp paired-end read data, and draft genome sequences were obtained successfully. Whole-genome sequencing (WGS) analysis provided comprehensive information for the *tet*(X)-carrying bacteria and their phylogenetic relationship. Multilocus sequence typing (MLST) analysis revealed that PK5074 positive for *tet*(X) and *mcr-1* belonged to sequence type 48 (ST48), and *tet*(X)-bearing strains PK5086 and PK5095 along with PK5097 belonged to ST410. We further determined the clonal relationship of strains PK5086, PK5095, and PK5097 based on their single nucleotide polymorphism (SNP) of the core genome. The numbers of differences in SNPs were only up to three between the three strains. In addition, PK5086, PK5095, and PK5097 contained identical antimicrobial resistance genes, insertion sequences, virulence-associated genes, and plasmid replicons (Fig. 1), indicating that clonal dissemination of *tet*(X)-positive strains existed in two different hospitals. Multiple antimicrobial resistance genes were identified in four isolates (Fig. 1).

To learn the genetic contexts of *tet*(X), PK5074 coharboring *tet*(X) and *mcr-1* and the representative PK5086 of the remaining three strains were sequenced with the Oxford Nanopore Technologies MinION long-read platform. PK5074 harbored a chromosome and five plasmids consisting of pPK5074-MCR1, pPK5074-tetX, pPK5074-91kb, pPK5074-69kb, and pPK5074-2kb (Table 2). The *tet*(X) gene was located on the IncFII plasmid pPK5074-tetX, which is an MDR plasmid coharboring *aph(3′)-Ia*, *aph(6)-Id*, *aadA22*, *aph(3′')-Ib*, *sul2*, *floR*, and *bla*_TEM-215_ genes (Table 2). pPK5074-MCR1 was a typical *mcr-1*-bearing IncHI2 plasmid carrying various resistance genes, including *mcr-1*, *aph(3′)-Ia*, *aadA8*, *lnu*(F), *sul3*, *tet*(A), *aph(6)-Id*, *floR*, *arr-2*, and *dfrA14* dispersed among insertion sequences (Table 2). PK5086 contained a chromosome and four plasmids consisting of pPK5086-tetX, pPK5086-97kb, pPK5086-95kb, and pPK5086-2kb (Table 2). The *tet*(X) gene was in plasmid pPK5086-tetX, which is also an MDR IncFII plasmid cocarrying *fosA4*, *mph*(A), *dfrA12*, *floR*, and *bla*_TEM-215_.

**FIG 2:**
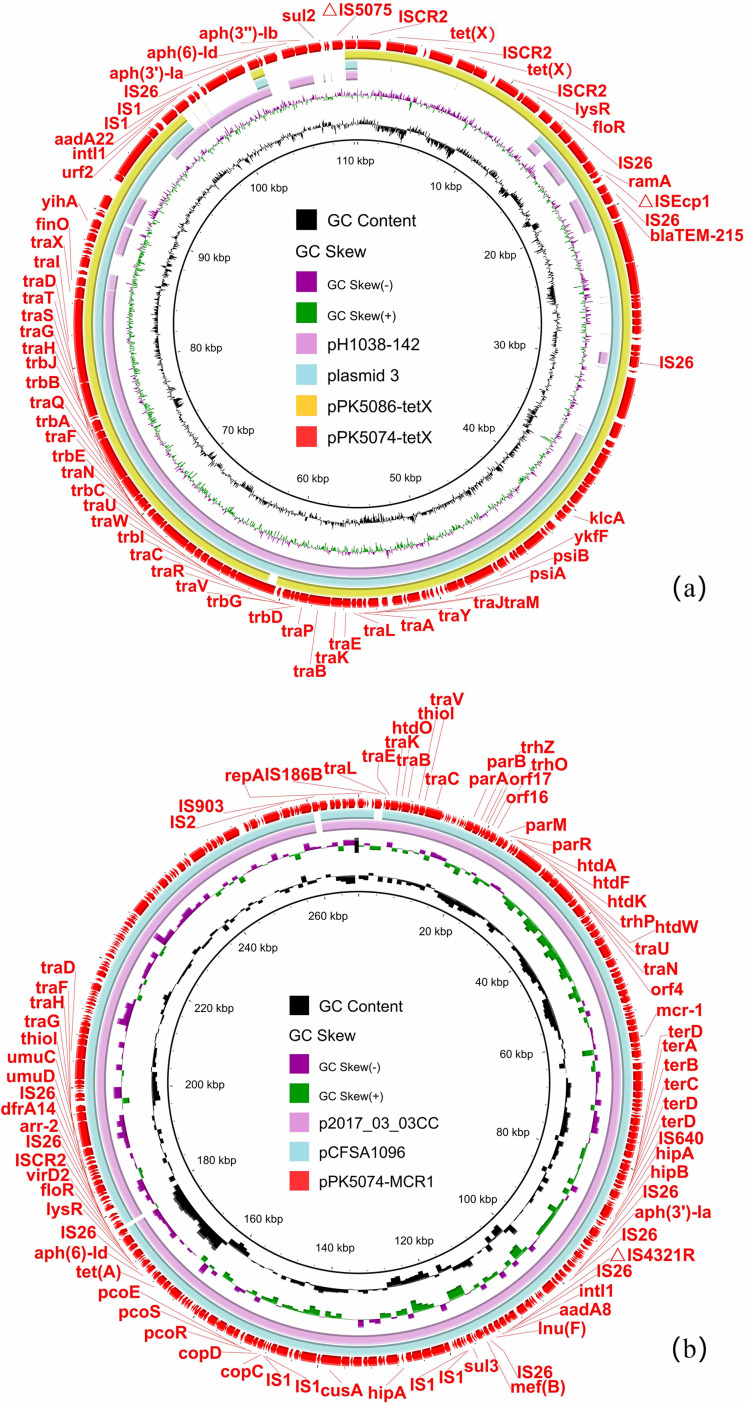
Sequence comparison of plasmids harboring *mcr-1* and *tet*(X) with structurally similar plasmids available in NCBI database. (a) Circular comparison of the *tet*(X)-bearing IncFII plasmids, including pPK5074-tetX, pPK5086-tetX, and other IncFII plasmids in the NCBI nr database. The outermost circle indicate reference plasmid pPK5074-tetX with genes annotated. (b) Circular comparison between the *mcr-1*-bearing IncHI2 plasmid pPK5074-MCR1 and other IncHI2 plasmids in the NCBI nr database.

BLASTn analysis of pPK5074-tetX and pPK5086-tetX against the NCBI nr database showed that they exhibited 99% identity at 78% coverage with plasmid 3 (LR130554) in E. coli MS14386 from a blood sample, and 97% identity at 64% coverage with plasmid pH1038-142 (KJ484634) in an E. coli isolate from a human (Fig. 2a). Plasmid 3 and pH1038-142 had plasmid backbone structures similar to those of pPK5074-tetX and pPK5086-tetX, but there was no MDR region including *tet*(X) in plasmid 3. The most obvious difference of pH1038-142, compared with pPK5074-tetX and pPK5086-tetX, was also an MDR region without *tet*(X) (Fig. 2a). This indicates that the formation of plasmid pPK5074-tetX and pPK5086-tetX may depend on the evolution of MDR regions. Two copies of IS*CR2* were adjacent to *tet*(X) in pPK5074-tetX (Fig. 2a), which may play a role in facilitating the transmission of *tet*(X) (3, 4). In addition, two repeats of *tet*(X) were found in pPK5074-tetX, and the repeat structure was IS*CR2*-*hp*-*abh*-*tet*(X) in 4,608 bp, which was the reported *tet*(X)-bearing circular intermediate (3, 6). The circular intermediate may play an important role in the formation of *tet*(X)-bearing tandem repeat structures. pPK5074-MCR1 shared >98% coverage and >99% identity with plasmid pCFSA1096 (CP033347) in Salmonella enterica subsp. *enterica* strain CFSA1096 of food origin in China and plasmid p2017_03_03CC (LC511658) in E. coli 2017.03.03CC isolate of human origin (Fig. 2b).

The isolate PK5074 belonged to the ST48 E. coli, which was linked to Shiga toxin-producing or extraintestinal pathogenic strains, and three *mcr-1*-carrying ST48 E. coli isolates were characterized as avian-pathogenic E. coli in Pakistan (7–9). Notably, ST48 strains were found to be a dominant host for the *mcr-1*-bearing IncX4 plasmid (10) and a host for the carbapenemase gene *bla*_NDM_ occasionally (11, 12). However, the *tet*(X) gene has also begun to appear in ST48 E. coli isolates, and this should attract our attention. In E. coli PK5074, pPK5074-tetX and pPK5074-MCR1 were MDR plasmids harboring various insertion sequences, such as IS*CR2* and IS*26* (Fig. 2a and b). It has been reported that IS*CR2* and IS*26* may facilitate the construction of large fused MDR plasmids (6, 13–15). Therefore, it is possible that the IncFII plasmid pPK5074-tetX and the IncHI2 plasmid pPK5074-MCR1 could form a recombinant plasmid carrying *tet*(X) and *mcr-1* mediated by insertion sequences. This will accelerate the transmission of *mcr-1* and *tet*(X), but the possibility warrants further investigations. In fact, the emergence of the plasmid-mediated tigecycline resistance gene *tet*(X) in E. coli isolated from poultry, food, and the environment in South Asia was reported in May 2021, and *tet*(X)-bearing IncFII or IncQ1 plasmid was found to coexist with *mcr-1*-carrying IncI2 plasmid (16). In combination with this study, we speculate that more *tet*(X)- and *mcr-1*-coharboring isolates will appear in the region and constitute a potential public health concern.

In isolate PK5086, the *tet*(X)-carrying plasmid pPK5086-tetX was highly similar to pPK5074-tetX except for the MDR region, and pPK5086-tetX harbored the transfer elements (Fig. 2a), but they were unable to transfer into J53 by conjugation. Given the high potential of ST410 E. coli to acquire resistance to last-resort antimicrobials (17), the establishment of *tet*(X)-carrying ST410 E. coli in South Asia should arouse regional and global concerns, as resistance to last-resort antibiotics is already a major public health crisis in the region and worldwide.

To conclude, we report the first identification of E. coli clinical isolates harboring *tet*(X) and *mcr-1* of human origin in Pakistan and report the cotransfer of *mcr-1*-bearing IncHI2 plasmid with *tet*(X)-positive plasmid in a clinical isolate. These findings indicate that mobile tigecycline and colistin resistance genes may disseminate in clinical settings in Pakistan and pose a serious global risk in clinical settings. It is recommended to strengthen the monitoring of the coexistence of *mcr-1* and *tet*(X) to avoid the coming of the preantibiotic era.

### Bacterial isolates and identification.

Between 2019 and 2020, a total of 100 human clinical isolates were screened for the presence of mobile tigecycline-resistant E. coli harboring *tet*(X) in Faisalabad, Pakistan. The human clinical E. coli isolates were collected from two tertiary care hospitals: 70 isolates were collected from hospital A and 30 isolates from hospital B. All isolates were cultivated on urinary tract infection (UTI) chrome agar supplemented with 2 mg/liter tigecycline and incubated overnight at 37°C for isolation of tigecycline-resistant E. coli strains. PCR was employed to screen the presence of *tet*(X) in tigecycline-resistant isolates using primers described earlier (3). *mcr-1* was further identified in *tet*(X)-positive isolates (18). 16S rRNA gene sequencing was performed to confirm bacterial species.

### Antimicrobial susceptibility testing.

The MICs of gentamicin, kanamycin, doxycycline, ampicillin, enrofloxacin, ceftiofur, streptomycin, amoxicillin, rifampin, ceftriaxone, florfenicol, meropenem, colistin, terramycin, tetracycline, and tigecycline for all *tet*(X)-bearing isolates were determined by the broth microdilution method in accordance with the Clinical and Laboratory Standards Institute (CLSI) guidelines (19) and were interpreted according to the CLSI standards (M100 and M31-A3) and the European Committee on Antimicrobial Susceptibility Testing (EUCAST) breakpoints (http://www.eucast.org/clinical_breakpoints/). Tigecycline and colistin were interpreted in accordance with the EUCAST guidelines (susceptible, ≤2 mg/liter; resistant, >2 mg/liter). E. coli ATCC 25922 served as the quality control strain.

### Conjugation experiments.

To investigate the transferability of *tet*(X) and *mcr-1*, conjugation assays were performed using *tet*(X)-positive strains as donors and E. coli J53 (sodium azide resistant [Azi^r^]) as the recipient. Bacterial strains were streaked onto LB agar plates, followed by inoculation into LB broth overnight. Cultures of donors and the recipient were mixed at 1:1, and then 0.1 ml of mixed culture was applied onto LB agar plates, followed by incubation at 37°C for 16 to 20 h. After incubation, we subsequently collected the mixed culture on LB agar plates and 10-fold serially diluted it in sterile saline. LB agar plates, supplemented with different antimicrobials, including tigecycline (2 mg/liter) and sodium azide (150 mg/liter), colistin (2 mg/liter) and sodium azide (150 mg/liter), and tigecycline in combination with colistin and sodium azide, were used to recover transconjugants [*tet*(X)-carrying, *mcr-1*-containing, and *tet*(X) and *mcr-1* coharboring transconjugants]. The presence of *tet*(X) or/and *mcr-1* genes in transconjugants was confirmed by PCR and antimicrobial susceptibility testing as described above. The frequency of conjugation transfer was calculated by the number of transconjugants per recipient as previously described (20).

### WGS and bioinformatics analysis.

The genomic DNA of all *tet*(X)-positive isolates was extracted using the FastPure bacteria DNA isolation minikit (Vazyme, China) in accordance with the manufacturer’s recommendations. Whole-genome sequencing was performed via an Illumina HiSeq 2500 platform, and two representative isolates were further sequenced by Oxford Nanopore Technologies (ONT) MinION platform. Short-read Illumina raw sequences were assembled using SPAdes (21). Illumina short-read and Nanopore long-read data were used to perform *de novo* assembly with Unicycler 0.4.4 (22, 23). The Rapid Annotation using Subsystems Technology annotation website server (https://rast.nmpdr.org/rast.cgi) was then used to annotate the genomes (24). Online tools, including PlasmidFinder 2.1 (25), ResFinder 4.1 (26), VirulenceFinder 2.0 (27), and MLST 2.0 (28), were utilized to assemble and characterize the genomes (https://cge.cbs.dtu.dk/services/). TBtools was used to visualize the distributions of antimicrobial resistance genes, insertion sequences, virulence-associated genes, and plasmid replicons (29). Comparisons of highly homologous complete plasmid sequences available in the NCBI database with plasmids in the study were performed with BRIG (30).

### Data availability.

The nucleotide sequences of the chromosomes and plasmids of E. coli PK5074 and PK5086 have been deposited in the NCBI database with accession numbers CP072802 to CP072807 and CP080370 to CP080374, respectively (Table 2). The draft genomes of E. coli PK5095 and PK5097 were also deposited in NCBI (BioProject identifier [ID] PRJNA751691).

## References

[1] Seifert H, Blondeau J, Dowzicky MJ. 2018. *In vitro* activity of tigecycline and comparators (2014–2016) among key WHO 'priority pathogens' and longitudinal assessment (2004–2016) of antimicrobial resistance: a report from the T.E.S.T. study. Int J Antimicrob Agents 52:474–484. doi:10.1016/j.ijantimicag.2018.07.003. 30012439

[2] Rodriguez-Bano J, Gutierrez-Gutierrez B, Machuca I, Pascual A. 2018. Treatment of infections caused by extended-spectrum-beta-lactamase-, AmpC-, and carbapenemase-producing *Enterobacteriaceae*. Clin Microbiol Rev 31:e00079-17. doi:10.1128/CMR.00079-17. 29444952 PMC5967687

[3] He T, Wang R, Liu D, Walsh TR, Zhang R, Lv Y, Ke Y, Ji Q, Wei R, Liu Z, Shen Y, Wang G, Sun L, Lei L, Lv Z, Li Y, Pang M, Wang L, Sun Q, Fu Y, Song H, Hao Y, Shen Z, Wang S, Chen G, Wu C, Shen J, Wang Y. 2019. Emergence of plasmid-mediated high-level tigecycline resistance genes in animals and humans. Nat Microbiol 4:1450–1456. doi:10.1038/s41564-019-0445-2. 31133751

[4] Sun J, Chen C, Cui CY, Zhang Y, Liu X, Cui ZH, Ma XY, Feng Y, Fang LX, Lian XL, Zhang RM, Tang YZ, Zhang KX, Liu HM, Zhuang ZH, Zhou SD, Lv JN, Du H, Huang B, Yu FY, Mathema B, Kreiswirth BN, Liao XP, Chen L, Liu YH. 2019. Plasmid-encoded *tet*(X) genes that confer high-level tigecycline resistance in *Escherichia coli*. Nat Microbiol 4:1457–1464. doi:10.1038/s41564-019-0496-4. 31235960 PMC6707864

[5] Ding Y, Saw WY, Tan LWL, Moong DKN, Nagarajan N, Teo YY, Seedorf H. 2020. Emergence of tigecycline- and eravacycline-resistant Tet(X4)-producing *Enterobacteriaceae* in the gut microbiota of healthy Singaporeans. J Antimicrob Chemother 75:3480–3484. doi:10.1093/jac/dkaa372. 32853333

[6] Li R, Lu X, Peng K, Liu Z, Li Y, Liu Y, Xiao X, Wang Z. 2020. Deciphering the structural diversity and classification of the mobile tigecycline resistance gene *tet*(X)-bearing plasmidome among bacteria. mSystems 5:e00134-20. doi:10.1128/mSystems.00134-20. 32345737 PMC7190383

[7] Furlan JPR, Gallo IFL, de Campos A, Passaglia J, Falcao JP, Navarro A, Nakazato G, Stehling EG. 2019. Molecular characterization of multidrug-resistant Shiga toxin-producing *Escherichia coli* harboring antimicrobial resistance genes obtained from a farmhouse. Pathog Glob Health 113:268–274. doi:10.1080/20477724.2019.1693712. 31757195 PMC6913633

[8] Zhuge X, Jiang M, Tang F, Sun Y, Ji Y, Xue F, Ren J, Zhu W, Dai J. 2019. Avian-source *mcr-1*-positive *Escherichia coli* is phylogenetically diverse and shares virulence characteristics with *E. coli* causing human extra-intestinal infections. Vet Microbiol 239:108483. doi:10.1016/j.vetmic.2019.108483. 31699469

[9] Azam M, Mohsin M, Johnson TJ, Smith EA, Johnson A, Umair M, Saleemi MK, Sajjad Ur R. 2020. Genomic landscape of multi-drug resistant avian pathogenic *Escherichia coli* recovered from broilers. Vet Microbiol 247:108766. doi:10.1016/j.vetmic.2020.108766. 32768218

[10] Wang Y, Liu H, Wang Q, Du X, Yu Y, Jiang Y. 2020. Coexistence of *bla*_KPC-2_-IncN and *mcr-1*-IncX4 plasmids in a ST48 *Escherichia coli* strain in China. J Glob Antimicrob Resist 23:149–153. doi:10.1016/j.jgar.2020.08.023. 32966910

[11] Liu Z, Wang Y, Walsh TR, Liu D, Shen Z, Zhang R, Yin W, Yao H, Li J, Shen J. 2017. Plasmid-mediated novel bla_NDM-17_ gene encoding a carbapenemase with enhanced activity in a sequence type 48 *Escherichia coli* strain. Antimicrob Agents Chemother 61:e02233-16. doi:10.1128/AAC.02233-16. 28242668 PMC5404555

[12] Liu Z, Xiao X, Li Y, Liu Y, Li R, Wang Z. 2019. Emergence of IncX3 plasmid-harboring *bla*_NDM-5_ dominated by *Escherichia coli* ST48 in a goose farm in Jiangsu, China. Front Microbiol 10:2002. doi:10.3389/fmicb.2019.02002. 31551956 PMC6737504

[13] Li R, Lu X, Peng K, Liu Y, Xiao X, Wang Z. 2020. Reorganization of *mcr-1*-bearing large MDR plasmids resolved by nanopore sequencing. J Antimicrob Chemother 75:1645–1647. doi:10.1093/jac/dkaa046. 32068848

[14] He S, Hickman AB, Varani AM, Siguier P, Chandler M, Dekker JP, Dyda F. 2015. Insertion sequence IS*26* reorganizes plasmids in clinically isolated multidrug-resistant bacteria by replicative transposition. mBio 6:e00762. doi:10.1128/mBio.00762-15. 26060276 PMC4471558

[15] He D, Zhu Y, Li R, Pan Y, Liu J, Yuan L, Hu G. 2019. Emergence of a hybrid plasmid derived from IncN1-F33:A-:B- and *mcr-1*-bearing plasmids mediated by IS*26*. J Antimicrob Chemother 74:3184–3189. doi:10.1093/jac/dkz327. 31360994

[16] Mohsin M, Hassan B, Martins W, Li R, Abdullah S, Sands K, Walsh TR. 2021. Emergence of plasmid-mediated tigecycline resistance *tet*(X4) gene in *Escherichia coli* isolated from poultry, food and the environment in South Asia. Sci Total Environ 787:147613. doi:10.1016/j.scitotenv.2021.147613. 33992939

[17] Nadimpalli ML, de Lauzanne A, Phe T, Borand L, Jacobs J, Fabre L, Naas T, Le Hello S, Stegger M. 2019. *Escherichia coli* ST410 among humans and the environment in Southeast Asia. Int J Antimicrob Agents 54:228–232. doi:10.1016/j.ijantimicag.2019.05.024. 31176748

[18] Rebelo AR, Bortolaia V, Kjeldgaard JS, Pedersen SK, Leekitcharoenphon P, Hansen IM, Guerra B, Malorny B, Borowiak M, Hammerl JA, Battisti A, Franco A, Alba P, Perrin-Guyomard A, Granier SA, De Frutos Escobar C, Malhotra-Kumar S, Villa L, Carattoli A, Hendriksen RS. 2018. Multiplex PCR for detection of plasmid-mediated colistin resistance determinants, *mcr-1*, *mcr-2*, *mcr-3*, *mcr-4* and *mcr-5* for surveillance purposes. Euro Surveill 23:17-00672. doi:10.2807/1560-7917.ES.2018.23.6.17-00672. PMC5824125 29439754

[19] CLSI. 2018. Performance standards for antimicrobial susceptibility testing, 28th ed. CLSI supplement M100. CLSI, Wayne, PA.

[20] Zhu W, Clark N, Patel JB. 2013. pSK41-like plasmid is necessary for Inc18-like *vanA* plasmid transfer from *Enterococcus faecalis* to *Staphylococcus aureus in vitro*. Antimicrob Agents Chemother 57:212–219. doi:10.1128/AAC.01587-12. 23089754 PMC3535934

[21] Bankevich A, Nurk S, Antipov D, Gurevich AA, Dvorkin M, Kulikov AS, Lesin VM, Nikolenko SI, Pham S, Prjibelski AD, Pyshkin AV, Sirotkin AV, Vyahhi N, Tesler G, Alekseyev MA, Pevzner PA. 2012. SPAdes: a new genome assembly algorithm and its applications to single-cell sequencing. J Comput Biol 19:455–477. doi:10.1089/cmb.2012.0021. 22506599 PMC3342519

[22] Li R, Xie M, Dong N, Lin D, Yang X, Wong MHY, Chan EW, Chen S. 2018. Efficient generation of complete sequences of MDR-encoding plasmids by rapid assembly of MinION barcoding sequencing data. Gigascience 7:1–9. doi:10.1093/gigascience/gix132. PMC5848804 29325009

[23] Wick RR, Judd LM, Gorrie CL, Holt KE. 2017. Unicycler: resolving bacterial genome assemblies from short and long sequencing reads. PLoS Comput Biol 13:e1005595. doi:10.1371/journal.pcbi.1005595. 28594827 PMC5481147

[24] Overbeek R, Olson R, Pusch GD, Olsen GJ, Davis JJ, Disz T, Edwards RA, Gerdes S, Parrello B, Shukla M, Vonstein V, Wattam AR, Xia F, Stevens R. 2014. The SEED and the Rapid Annotation of microbial genomes using Subsystems Technology (RAST). Nucleic Acids Res 42:D206–D214. doi:10.1093/nar/gkt1226. 24293654 PMC3965101

[25] Carattoli A, Zankari E, Garcia-Fernandez A, Voldby Larsen M, Lund O, Villa L, Moller Aarestrup F, Hasman H. 2014. *In silico* detection and typing of plasmids using PlasmidFinder and plasmid multilocus sequence typing. Antimicrob Agents Chemother 58:3895–3903. doi:10.1128/AAC.02412-14. 24777092 PMC4068535

[26] Bortolaia V, Kaas RS, Ruppe E, Roberts MC, Schwarz S, Cattoir V, Philippon A, Allesoe RL, Rebelo AR, Florensa AF, Fagelhauer L, Chakraborty T, Neumann B, Werner G, Bender JK, Stingl K, Nguyen M, Coppens J, Xavier BB, Malhotra-Kumar S, Westh H, Pinholt M, Anjum MF, Duggett NA, Kempf I, Nykasenoja S, Olkkola S, Wieczorek K, Amaro A, Clemente L, Mossong J, Losch S, Ragimbeau C, Lund O, Aarestrup FM. 2020. ResFinder 4.0 for predictions of phenotypes from genotypes. J Antimicrob Chemother 75:3491–3500. doi:10.1093/jac/dkaa345. 32780112 PMC7662176

[27] Joensen KG, Scheutz F, Lund O, Hasman H, Kaas RS, Nielsen EM, Aarestrup FM. 2014. Real-time whole-genome sequencing for routine typing, surveillance, and outbreak detection of verotoxigenic *Escherichia coli*. J Clin Microbiol 52:1501–1510. doi:10.1128/JCM.03617-13. 24574290 PMC3993690

[28] Larsen MV, Cosentino S, Rasmussen S, Friis C, Hasman H, Marvig RL, Jelsbak L, Sicheritz-Ponten T, Ussery DW, Aarestrup FM, Lund O. 2012. Multilocus sequence typing of total-genome-sequenced bacteria. J Clin Microbiol 50:1355–1361. doi:10.1128/JCM.06094-11. 22238442 PMC3318499

[29] Chen C, Chen H, Zhang Y, Thomas HR, Frank MH, He Y, Xia R. 2020. TBtools: an integrative toolkit developed for interactive analyses of big biological data. Mol Plant 13:1194–1202. doi:10.1016/j.molp.2020.06.009. 32585190

[30] Alikhan NF, Petty NK, Ben Zakour NL, Beatson SA. 2011. BLAST Ring Image Generator (BRIG): simple prokaryote genome comparisons. BMC Genomics 12:402. doi:10.1186/1471-2164-12-402. 21824423 PMC3163573

